# Detection and characterization of two chimpanzee polyomavirus genotypes from different subspecies

**DOI:** 10.1186/1743-422X-7-347

**Published:** 2010-11-26

**Authors:** Ilona Deuzing, Zahra Fagrouch, Marlous J Groenewoud, Henk Niphuis, Ivanela Kondova, Willy Bogers, Ernst J Verschoor

**Affiliations:** 1 Department of Virology, Biomedical Primate Research Centre (BPRC), Rijswijk, The Netherlands; 2 Animal Science Department, Biomedical Primate Research Centre (BPRC), Rijswijk, The Netherlands; 3 Department of Virology, Erasmus University Medical Center, Rotterdam, The Netherlands; 4 Department of Physiological Chemistry and Centre for Biomedical Genetics, University Medical Center, Utrecht, The Netherlands

## Abstract

The complete nucleotide sequences of three chimpanzee polyomavirus genetic variants were determined. Phylogenetic analysis indicated that the viruses form two different genotypes of ChPyV. Comparison with other primate polyomaviruses revealed a putative agnogene, and an unusually long VP1 open reading frame. The transcriptional control regions (TCR) of the viruses were extremely short (155 nucleotides), and highly conserved amongst the genotypes. Analysis of the TCR from different chimpanzee subspecies, and from a series of tissues from five individuals confirmed its genetic stability, and also indicates that double-infections with different genotypes can occur.

## Findings

The number of primate polyomaviruses (PyV), including human polyomaviruses, has rapidly expanded in recent years. Six human viruses, KIPyV, WUPyV, Merkel cell polyomavirus (McPyV), Trichodysplasia Spinulosa-associated polyomavirus (TSPyV), HPyV6, and HPyV7 have been characterized in patients suffering from respiratory tract infections (KI and WU), Merkel cell carcinomas (MC), virus-associated trichodysplasia spinulosa (TSP), or were detected in the skin of healthy individuals (HPyV6, and HPyV7) [1-5]. Simultaneously, novel simian viruses have been discovered in healthy squirrel monkeys and orangutans [6,7], and in diarrheal stool from a chimpanzee [8]. From the chimpanzee polyomavirus (ChPyV) only the nucleotide sequence of the VP1 gene has been published [GenBank: AY691168]. We have investigated the genetic variation of ChPyV, and sequenced the genome of three chimpanzee polyomavirus variants. We also analyzed the genetic variation of ChPyV in relation to the host subspecies, and investigated ChPyV tissue tropism.

**Figure 1:**
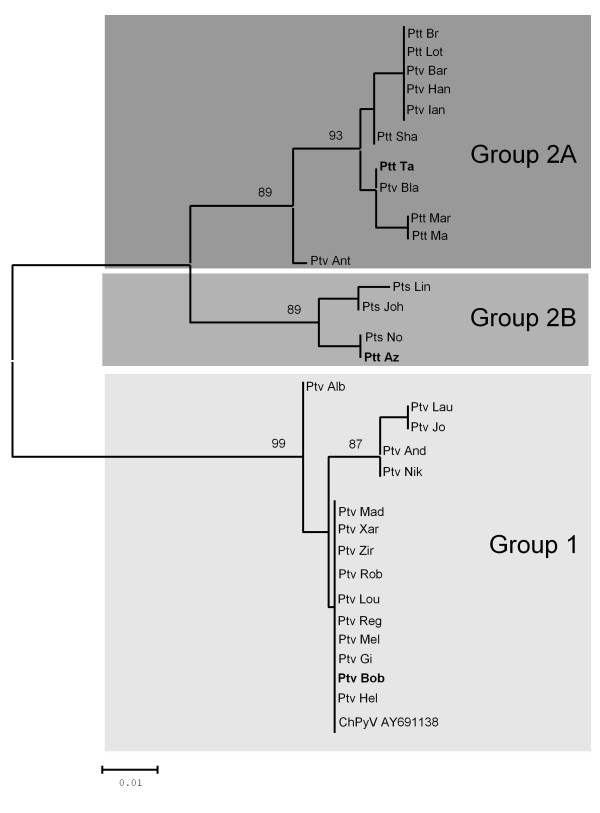
**Phylogenetic tree constructed using partial VP1 gene sequences of chimpanzee polyomaviruses**. Grey shading indicates genogroups described in the text, and isolates used for complete genome sequencing are in bold. The published ChPyV VP1 sequence (AY691138) is included in the tree. Sequence alignments were made by using MacVector version 10.6. Phylogenetic analysis was performed by the Neighbor-Joining method as implemented in MEGA version 4 [25]. Bootstrap values (as % of 1000 re-samplings) are indicated. Bar, 0.01 nucleotide replacements per site. First three letters of name indicate subspecies: Ptv, *Pan troglodytes verus*; Ptt, *P.t. troglodytes*; Pts, *P.t. schweinfurthii*. [EMBL: FR692245-FR692275].

ChPyV VP1-specific PCR primers, based on the published VP1 sequence, were used to screen DNA isolated from blood samples collected from captive and wild-caught chimpanzees (QIAamp DNA Mini Kit, QIAGEN Benelux BV, Venlo, The Netherlands) (Table 1). Captive animals originated from former chimpanzee colonies kept at the BPRC (n = 66) and another primate facility in Europe (n = 24). Materials from wild-caught chimpanzees were obtained from animals housed in a rehabilitation centre in Africa (n = 22). The outer amplification reaction was performed in a 50 μl volume using 1 μg of DNA, 2 units Maxima™ Hot Start *Taq *DNA polymerase (Fermentas GMBH, St. Leon-Rot, Germany), 5 μl 10 × Hot Start PCR buffer, 1 pmol of each primer, 2 mM MgCl_2_, and 200 μM of each dNTP. Cycling conditions for both reactions were 95°C for 30 sec, 55°C for 30 sec, and 72°C for 30 sec. In a second amplification reaction, 2 μl of the PCR product of the outer PCR was used as template. Inner PCR conditions were identical to those for the outer PCR, except that 2.5 mM MgCl_2 _was used. The PCR fragments were gel-purified using the Zymoclean™ Gel DNA Recovery Kit (Zymo Research Corp, Orange, USA), and sequence analysis was performed directly on the purified amplicons (Baseclear BV, Leiden, The Netherlands). Thirty VP1 sequences were obtained and sequenced, and phylogenetic analysis revealed the presence of two genetic groups, one of which consisted of two smaller subclusters (genogroup 2A and 2B; Figure 1). We next investigated if there was a relationship between viral genotype and chimpanzee subspecies. The chimpanzee subspecies was determined by analysis of mitochondrial control region (D-loop) [9], and data showed that genogroup 1 solely consisted of viruses derived from individuals belonging to the *Pan troglodytes verus *subspecies, while group 2 was formed by viruses obtained from the three major subspecies *Pt. verus*, *Pt. troglodytes*, and *Pt. schweinfurthii*.

**Table 1:** Primers used for PCR amplification of VP1 and TCR sequences

Primer name	Sequence (5' > 3')
**ChPyV VP1 assay**	

ChPyV-Fout	GTTATTCATCATGCAGATGG

ChPyV-Rout	TCAGCTAATTTAGCTATATC

ChPyV-Fin	GAACACAGACATGACCTGTG

ChPyV-Rin	GTATAGCTGAAGCATATTTAG

**ChPyV TCR assay**	

TCRoutF	AAAGTTTTACATCATAGCAATCAGA

TCRoutR	AGAGGGCTTCAATAGTCAATCCAGA

TCRinF	GACCCTCTTGAAATTTTTGCCACAGT

TCRinR	TTAGTTCAGAAGCCATCACAATCATA

**Figure 2:**
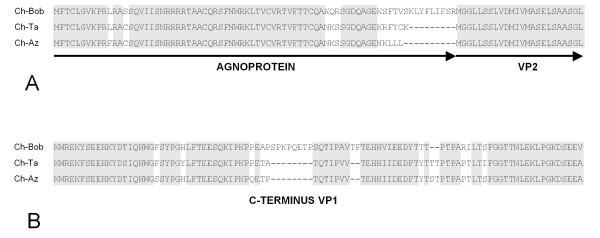
**Alignments of chimpanzee polyomavirus proteins.** A. Comparison of the agnoprotein-VP2 junction of ChPyV variants. The putative agnoproteins and the N-terminal 24 amino acid residues of VP2 are aligned. Areas with similarities and identities within the three agnoprotein-VP2 junctions are shaded grey. B. Alignment of the C-terminus of the chimpanzee polyomavirus VP1 proteins. Areas with similarities and identities within the three VP1 proteins are shaded grey.

Full-length nucleotide sequences of representatives from each variant were determined using long-distance PCR [6,7]. Sequence comparison of the genomes confirmed that two variants ChPyV-Ta and -Az (genogroup 2A and 2B, respectively) were more similar to each other (96.6%), than to ChPyV-Bob, a genogroup 1 virus (92.6% and 92.7%, respectively). Sequences have been deposited under EMBL database accession numbers FR692334 to FR692336. Further analysis confirmed a typical polyomavirus genetic structure of each variant, with an early region encoding the small t- (t-Ag) and large T-antigens (T-Ag), and a late region encoding the VP1, VP2, and VP3 structural proteins. All three viruses accommodate a potential agnogene, encoding a protein of 64, 65, or 74 amino acids for ChPyV-Az, ChPyV-Ta and ChPyV-Bob, respectively. The first two agnogenes are located 5' to the VP2/VP3 open reading frame (orf), but curiously the agnogene of ChPyV-Bob is fused in-frame with the VP2/VP3 orf. An alignment of the agno-VP2 junction, illustrating the disparity between the viral genomes, is given in Figure 2A. The VP1 structural proteins encoded by the ChPyV genomes are considerably longer than VP1 from other polyomaviruses. The VP1 orf of ChPyV-Bob (nt. 1033-2526) encodes a protein of 498 amino acid residues (aa.) that has an additional 75 amino acids at its C-terminus compared to the longest VP1 described to date, that of the McPyV. Within the same C-terminus of ChPyV-Az and -Ta, an 8 aa. deletion (nt. 2356-2380) is found (Figure 2B). BLAST analysis of this region did not reveal any similarity with other known proteins. Search for specific polypeptide motifs or patterns (ExPASy proteomics server; http://www.expasy.ch/tools/) was also unsuccessful. The amino acid sequence similarity of the ChPyV structural proteins (represented by ChPyV-Ta) with known human and simian polyomaviruses is shown in table 2. Strikingly, within the early region the highest similarity is found with t-Ag and T-Ag from the human Merkel cell polyomavirus, while the late proteins, VP1- VP3, are most similar to the equivalent proteins of the polyomavirus from Sumatran orangutans.

**Table 2:** Protein sequence similarity (%) between ChPyV-Az and known primate polyomaviruses.

	JCV	KIPyV	McPyV	TSPyV	HPyV6	OraPyV-Sum	LPV	SquiPyV
**VP1**	44,2	30,4	**54,2**	53,9	34,0	51,9	51,1	47,3

**VP2**	31,8	21,5	**58,0**	43,2	24,5	44,0	35,7	33,3

**VP3**	26,7	19,2	**41,7**	25,6	21,6	26,0	22,3	25,0

**t-Ag**	40,2	39,3	47,0	40,2	42,4	**49,8**	45,8	40,0

**T-Ag**	49,7	53,3	50,5	58,8	54,6	**59,5**	57,5	54,5

Proteins with highest percentage similarity with ChPyV proteins are given in bold italic.

**Figure 3:**
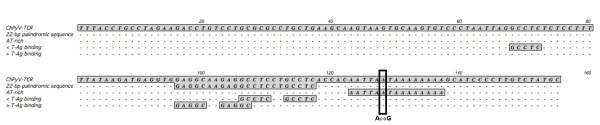
**Architecture of the ChPyV transcriptional control region**. The > indicate the direction T-Ag binding site. The variable nucleotide 128 is boxed.

The transcriptional control region (TCR) of polyomaviruses controls gene expression and viral replication. This region, located between the start of the t-Ag orf, and the start of the putative agnoprotein orf, is only 155 bp long for all three ChPyV variants, and is the shortest TCR of all PyV presently known. It is practically conserved between the viral variants; the TCR of ChPyV-Bob differs only at nucleotide 128 with the other TCRs. Consequently, the architecture of the TCR is simple (Figure 3). A 22-bp palindromic sequence is located at nt. 96-117, and contains two tandemlypositioned T-ag binding sites. An additional binding site is found at nt. 68-72, and is directed towards the early region. In contrast to other polyomavirus TCRs no repeated sequences are distinguishable. This feature makes the ChPyV the most basic TCR yet characterized, exceeding the proto-archetypal SV40 TCR in simplicity [10-12]. The SV40 TCR is a highly variable region that is mainly due to extensive rearrangements of enhancer elements caused by propagation of the virus in cell culture [13,14]. Evidence also indicates that rearrangements play a role in viral pathogenesis [15,16], and, recently it was found that in kidney transplant recipients a re-arranged TCR conferred BKV with a higher replicating capacity [17]. From a group of 23 animals, consisting of 16 *Pt. verus*, 6 *Pt. troglodytes*, and one *Pt. schweinfurthii*, the TCR region was amplified in a nested PCR assay (Table 1). PCR mixes were identical to the VP1 assay, except that 2 mM MgCl_2 _was used. Amplification conditions were: an enzyme activation step of 4 min at 96°C, followed by 40 amplification cycles of 95°C for 30 sec, 55°C for 30 sec, and 72°C for 45 sec. Sequence analysis revealed minimal TCR variation was observed [EMBL: FR692222-FR692244]. In 13 animals, an adenine instead of a guanine was seen at nucleotide 128, which is located within the AT-rich region. Of interest, all animals that had the adenine at this position belonged to the *Pt. verus *subspecies.

We also investigated the presence of ChPyV in different tissues taken at autopsy from five *Pt. verus *chimpanzees. The animals, varying from 7 to 43 years old, died of various causes. Histopathological examination did not reveal any lesions related to polyomavirus infection, like interstitial lymphoplasmacytic nephritis with occasional epithelial intranuclear inclusion bodies, proliferative interstitial pneumonia with intranuclear inclusions within type II pneumocytes, areas of demyelination of subcortical white matter, and/or intranuclear inclusions within astrocytes and oligodendrocytes (typical for progressive multifocal leucoencephalopathy; PML). The findings, as well as the cause of dead and the age are summarized in Table 3. All tissue samples were screened with the VP1 and TCR assays. An overview of the tissues analyzed from each individual, and PCR results is given in Table S1 (Additional file 1). Although the type and number of tissues analyzed from each animal varied considerable, it was evident that virus tissue distribution in Regina was most widespread. Regina was a 42-year-old female who was euthanized because of deteriorating body condition. The virus was easily detectable in 31 of 35 tissues tested, including the skin, and was undetectable in only a few tissues (parotid, muscle, aorta and sciatic nerve). Notably, the same tissues from the other chimpanzees were also negative, with the exception of the sciatic nerve sample from Antoine, which was positive in the TCR test, but not in the VP1 assay. Recent data from human polyomaviruses point to the skin as a target organ for PyV persistence and replication [2,4,5]. Interestingly, the skin was positive in all samples (n = 4) that were analyzed from our chimpanzees. In Melanie and Gina, both chimpanzees with a low number of tissues infected (4 of 32, and 4 of 33, respectively), the skin belonged to the few PCR-positive tissues. This suggests a similar skin tropism for ChPyV as for the human viruses. In addition, in 4 of 5 spleen samples the virus was easily detectable by PCR, while a more generally accepted target organ like the kidney, scored only 1 out of 5 DNA samples positive.

**Table 3:** Animals examined in this study

Animal	Age	Cause of dead	Histopathological findings
Regina	42 y	Euthanised because of deteriorating body condition	Mostly age related lesions: focal endocarditis; chronic interstitial nephritis; myodegeneration; mild lymphoid depletion in spleen No polyomavirus-associated lesions

Gina	43 y	Drowned	Bronchopneumonia; No polyomavirus-associated lesions

Melanie	12 y	Severely emaciated; died during anesthesia	Subacute pneumonia; hemosiderosis (spleen, liver)

Antoine	7 y	Died during anesthesia	No polyomavirus-associated lesions

Bob	14 y	Euthanised after episode of severe hematuria	Immune-mediated hemolytic anemia; No polyomavirus-associated lesions

The TCR of all 32 positive tissue samples was sequenced [EMBL: FR692190-FR692221]. Variation was minimal and similar to the abovementioned results in animals from different origin. In 25 TCR sequences (1 from Bob, 2 from Gina and Melanie, and 20 from Regina) an adenosine was identified at nucleotide 128, while in all 6 sequences from Antoine, and in 1 out of 2 TCRs obtained from Bob a guanine was found at this site. This strongly suggests that Bob was double infected with two viral variants, although a point mutation, occurred during viral replication, cannot be completely ruled out.

**Figure 4:**
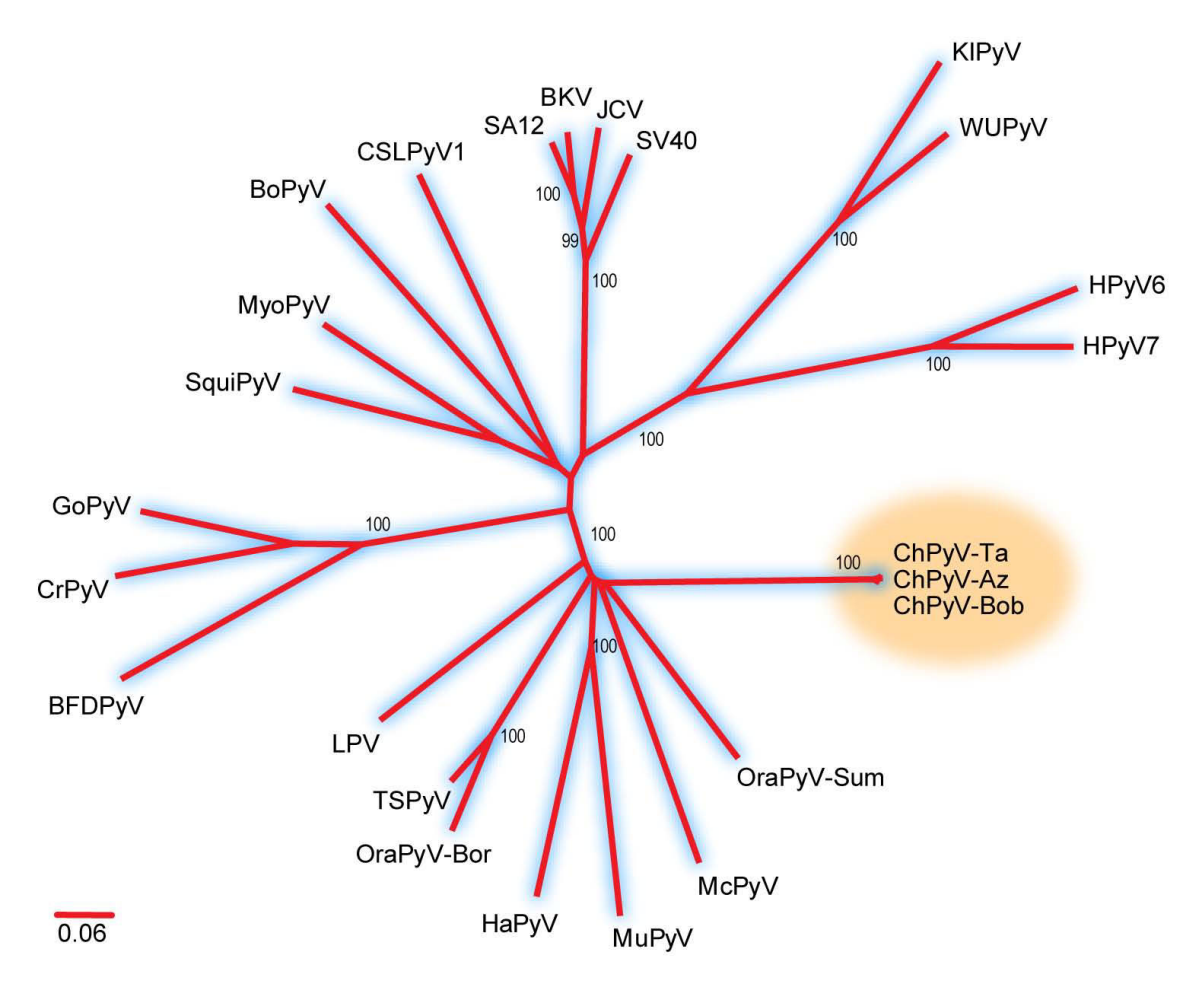
**Phylogenetic analysis of concatenated VP1 and Large T proteins from avian and mammalian polyomaviruses**. Sequence alignments were made by using MacVector version 10.6. The GapStreeze program (Los Alamos HIV Sequence Database; http://www.hiv.lanl.gov/content/sequence/GAPSTREEZE/gap.html) was used to remove columns with a gap tolerance of 0%. Phylogenetic analysis was performed by the neighbor-joining method using the JTT matrix model as implemented in MEGA version 4 [25]. Bootstrap values (as % of 1000 re-samplings) are indicated. Bar, 0.06 amino acid residue replacements per site. The GenBank accession numbers of the viruses used are: NC_001515 (MuPyV), NC_001663 (HaPyV), HM355825 (McPyV), M30540 (LPV), NC_001442 (BoPyV), NC_009951 (SquiPyV), NC_011310 (MyoPyV), NC_007922 (CrPyV), NC_004800 (GoPyV), AB453166 (BFDPyV), NC_001669 (SV40), NC_001699 (JCV), AY614708 (SA12), NC_001538 (BKV), EF127906 (KIPyV), EF444549 (WUPyV), FN356900 (OraPyV-Bor), FN356901 (OraPyV-Sum), GU989205 (TSPyV), NC_014407 (HPyV6), NC_014407 (HPyV7), NC_013796 (CSLPyV1). Chimpanzee polyomaviruses are highlighted.

In this study we have molecularly characterized three variants of the chimpanzee polyomavirus, and took a glimpse at some biological and evolutionary properties of this virus. Phylogenetic analysis of the concatenated VP1 and T-Ag protein sequences from avian and mammalian polyomaviruses show that the chimpanzee viruses form a distinct group of viruses, distantly related to the human McPyV and TSPyV, the orangutan polyomaviruses and LPV from African green monkeys. Interestingly, both rodent viruses (MuPyV and HaPyV) also fall within this large cluster (Figure 4) The chimpanzee polyomavirus genomes have some unique features, as they encode for unusually long VP1 structural proteins, and, in contrast, possess an exceptionally short TCR. The exact significance of these finding needs to be substantiated, and goes beyond the scope of this paper. Most interesting is the short and conserved TCR of the chimpanzee virus. Because the polyomavirus TCR regulates viral replication and pathogenesis, and its sequence variation in other PyV is likely the cause or consequence of these processes [15-17], it is an intriguing question how ChPyV with such a 'basic' and apparently genetically constant TCR regulates these processes. Our findings add to the increasing awareness that the *Polyomaviridae *are a genetically diverse family of viruses. In a recent study, van der Meijden *et al*. distinguished seven PyV clades, and pointed towards a complex evolutionary history [5]. The number of PyV has increased in the last few years; viruses have been detected in Californian sea lions (CSLPyV1) [18], bats (MyoPyV) [19], birds [20,21], in addition to the novel simian viruses. We have detected new polyomaviruses in apes (gorillas and bonoboos), Old World monkeys, like hamadryas baboon and mandrill, and in capuchin monkeys and spider monkeys, both New World monkeys (unpublished data; EMBL: FR692182-FR692189). With the help of improved diagnostic techniques and the use of metagenomic approaches [22-24] it can be expected that more polyomaviruses will be detected in the near future.

## Abbreviations

PyV: polyomavirus; TCR: transcriptional control region.

## Competing interests

The authors declare that they have no competing interests.

## Authors' contributions

ID and ZF contributed in obtaining PCR data and sequencing. HN provided chimpanzee blood samples. MJG performed long PCR and genome sequencing. IK was responsible for histopathological analysis and provided tissue samples. WB was helpful in interpreting the data. EJV was responsible for the planning of the study, data analysis, and drafted the manuscript. All authors have read and approved the final manuscript

## Supplementary Material

Additional file 1 **Table S1**. PCR analysis of chimpanzee tissues. Click here for file

## References

[B1] Allander T Andreasson K Gupta S Bjerkner A Bogdanovic G Persson MAA Dalianis T Ramqvist T Andersson B Identification of a third human polyomavirus J Virol 2007 81 4130 4136 10.1128/JVI.00028-07 17287263 PMC1866148

[B2] Feng H Shuda M Chang Y Moore PS Clonal integration of a polyomavirus in human Merkel cell carcinoma Science 2008 319 1096 1100 10.1126/science.1152586 18202256 PMC2740911

[B3] Gaynor AM Nissen MD Whiley DM Mackay IM Lambert SB Wu G Brennan DC Storch GA Sloots TP Wang D Identification of a novel polyomavirus from patients with acute respiratory tract infections PLoS Pathogens 2007 3 e64 10.1371/journal.ppat.0030064 17480120 PMC1864993

[B4] Schowalter RM Pastrana DV Pumphrey KA Moyer AL Buck CB Merkel cell polyomavirus and two previously unknown polyomaviruses are chronically shed from human skin Cell Host Microbe 2010 7 509 515 10.1016/j.chom.2010.05.006 20542254 PMC2919322

[B5] van der Meijden E Janssens RW Lauber C Bouwes Bavinck JN Gorbalenya AE Feltkamp MC Discovery of a new human polyomavirus associated with trichodysplasia spinulosa in an immunocompromized patient PLoS Pathog 2010 6 e1001024 10.1371/journal.ppat.1001024 20686659 PMC2912394

[B6] Groenewoud MJ Fagrouch Z van Gessel S Niphuis H Bulavaite A Warren KS Heeney JL Verschoor EJ Characterization of novel polyomaviruses from Bornean and Sumatran orang-utans J Gen Virol 2010 91 653 658 10.1099/vir.0.017673-0 19923267

[B7] Verschoor EJ Groenewoud MJ Fagrouch Z Kewalapat A van Gessel S Kik MJ Heeney JL Molecular characterization of the first polyomavirus from a New World primate: squirrel monkey polyomavirus J Gen Virol 2008 89 130 137 10.1099/vir.0.83287-0 18089736

[B8] Johne R Enderlein D Nieper H Muller H Novel polyomavirus detected in the feces of a chimpanzee by nested broad-spectrum PCR J Virol 2005 79 3883 3887 10.1128/JVI.79.6.3883-3887.2005 15731285 PMC1075742

[B9] de Groot NG Garcia CA Verschoor EJ Doxiadis GG Marsh SG Otting N Bontrop RE Reduced MIC gene repertoire variation in West African chimpanzees as compared to humans Mol Biol Evol 2005 22 1375 1385 10.1093/molbev/msi127 15758205

[B10] Lednicky JA Butel JS Consideration of PCR methods for the detection of SV40 in tissue and DNA specimens Dev Biol Stand 1998 94 155 164 9776238

[B11] Lednicky JA Butel JS Simian virus 40 regulatory region structural diversity and the association of viral archetypal regulatory regions with human brain tumors Semin Cancer Biol 2001 11 39 47 10.1006/scbi.2000.0345 11243898

[B12] White MK Safak M Khalili K Regulation of gene expression in primate polyomaviruses J Virol 2009 83 10846 10856 10.1128/JVI.00542-09 19640999 PMC2772795

[B13] Lednicky JA Butel JS Tissue culture adaptation of natural isolates of simian virus 40: changes occur in viral regulatory region but not in carboxy-terminal domain of large T-antigen J Gen Virol 1997 78 Pt 7 1697 1705 9225047 10.1099/0022-1317-78-7-1697

[B14] O'Neill FJ Greenlee JE Carney H The archetype enhancer of simian virus 40 DNA is duplicated during virus growth in human cells and rhesus monkey kidney cells but not in green monkey kidney cells Virology 2003 310 173 182 10.1016/S0042-6822(03)00116-8 12788641

[B15] Gosert R Rinaldo CH Funk GA Egli A Ramos E Drachenberg CB Hirsch HH Polyomavirus BK with rearranged noncoding control region emerge in vivo in renal transplant patients and increase viral replication and cytopathology J Exp Med 2008 205 841 852 10.1084/jem.20072097 18347101 PMC2292223

[B16] Yogo Y Zhong S Shibuya A Kitamura T Homma Y Transcriptional control region rearrangements associated with the evolution of JC polyomavirus Virology 2008 380 118 123 10.1016/j.virol.2008.07.016 18718622

[B17] Olsen GH Hirsch HH Rinaldo CH Functional analysis of polyomavirus BK non-coding control region quasispecies from kidney transplant recipients J Med Virol 2009 81 1959 1967 10.1002/jmv.21605 19774689

[B18] Wellehan JF Jr Yu F Venn-Watson SK Jensen ED Smith CR Farmerie WG Nollens HH Characterization of San Miguel sea lion virus populations using pyrosequencing-based methods Infect Genet Evol 2010 10 254 260 10.1016/j.meegid.2009.11.013 19931646 PMC7106084

[B19] Misra V Dumonceaux T Dubois J Willis C Nadin-Davis S Severini A Wandeler A Lindsay R Artsob H Detection of polyoma and corona viruses in bats of Canada J Gen Virol 2009 90 2015 2022 10.1099/vir.0.010694-0 19357225

[B20] Arroube AS Halami MY Johne R Dorrestein GM Mortality due to polyomavirus infection in two nightjars (Caprimulgus europaeus) J Avian Med Surg 2009 23 136 140 10.1647/2008-007.1 19673460

[B21] Halami MY Dorrestein GM Couteel P Heckel G Muller H Johne R Whole genome characterization of a novel polyomavirus detected in fatally diseased canary birds J Gen Virol 2010 91 3016 3022 10.1099/vir.0.023549-0 20797969

[B22] Ambrose HE Clewley JP Virus discovery by sequence-independent genome amplification Rev Med Virol 2006 16 365 383 10.1002/rmv.515 16929467 PMC7169205

[B23] Finkbeiner SR Allred AF Tarr PI Klein EJ Kirkwood CD Wang D Metagenomic analysis of human diarrhea: viral detection and discovery PLoS Pathog 2008 4 e1000011 10.1371/journal.ppat.1000011 18398449 PMC2290972

[B24] Li L Victoria JG Wang C Jones M Fellers GM Kunz TH Delwart E Bat guano virome: predominance of dietary viruses from insects and plants plus novel mammalian viruses J Virol 2010 84 6955 6965 10.1128/JVI.00501-10 20463061 PMC2898246

[B25] Tamura K Dudley J Nei M Kumar S MEGA4: Molecular Evolutionary Genetics Analysis (MEGA) software version 4.0 Mol Biol Evol 2007 24 1596 1599 10.1093/molbev/msm092 17488738

