# Supplementary material for: Detection and characterization of two chimpanzee polyomavirus genotypes from different subspecies
Source: Virol J. 2010 Nov 26;7:347. doi: 10.1186/1743-422X-7-347 (PMC3003640; doi:10.1186/1743-422X-7-347)
Supplement: Additional file 1 — Table S1. PCR analysis of chimpanzee tissues. [file 1743-422X-7-347-S1.DOC]

**Table S1. PCR analysis of chimpanzee tissues**

|  | **Regina** | **Gina** | | **Melanie** | | **Antoine** | | **Bob** | |
| --- | --- | --- | --- | --- | --- | --- | --- | --- | --- |
| **Tissue** | **VP1/TCR** | | **VP1/TCR** | | **VP1/TCR** | | **VP1/TCR** | | **VP1/TCR** |
| Thymus | +/+ | |  | | +/+ | | -/- | |  |
| Tonsil | +/- | | -/- | | -/- | |  | |  |
| Mes. LN | +/- | | -/- | | -/+ | |  | |  |
| Ing. LN | +/+ | | -/- | | -/- | | +/- | |  |
| Ax. LN | +/+ | | -/- | | -/- | | -/- | | -/- |
| Spleen | +/+ | | +/- | | -/- | | +/+ | | +/+ |
| Lung | +/+ | | -/- | | -/- | | +/+ | | -/- |
| Tongue | +/+ | | -/- | | -/- | |  | |  |
| Parotid | -/- | | -/- | | -/- | |  | |  |
| Submandibular gland | +/+ | | -/- | | -/- | |  | |  |
| Esophagus | +/+ | | -/- | | -/- | |  | |  |
| Stomach (cardia) | -/+ | | -/- | | -/- | | +/- | | -/- |
| Pancreas | +/- | | -/- | | -/- | | -/- | | -/- |
| Liver | +/+ | | -/- | | -/- | | +/- | | -/- |
| Gall bladder | +/+ | | -/- | | -/- | |  | |  |
| Duodenum | +/+ | | -/- | | -/- | |  | |  |
| Jejunum | -/+ | | -/- | | -/- | |  | | +/- |
| Ileum | +/+ | | -/- | | -/- | |  | |  |
| Colon | +/- | | -/- | | -/- | | +/- | | -/- |
| Caecum | +/- | | +/- | | -/- | |  | |  |
| Kidney | +/+ | | -/- | | -/- | | -/- | | -/- |
| Adrenal gland | +/- | | -/- | | -/- | | -/- | |  |
| Urine bladder | +/- | | -/- | | -/- | | +/- | | -/- |
| Ureter (left) | +/+ | |  | |  | |  | |  |
| Skin | -/+ | | +/+ | | +/- | | +/+ | |  |
| Muscle | -/- | | -/- | | -/- | | -/- | |  |
| Aorta | -/- | | -/- | | -/- | |  | |  |
| Aorta abd. |  | |  | | +/- | |  | |  |
| Aorta thor. |  | |  | | -/- | |  | |  |
| Heart | +/+ | | -/- | | -/- | | +/+ | | -/- |
| Pericard | +/- | | -/- | | -/- | |  | |  |
| Thyroid gland | +/+ | | -/- | | -/- | |  | |  |
| Brain | +/- | |  | |  | |  | | -/+ |
| Sciatic nerve | -/- | | -/- | | -/- | | -/+ | |  |
| Testis | NA | | NA | | NA | | +/+ | | +/- |
| Cervix |  | | -/- | |  | | NA | | NA |
| Uterus | -/+ | | -/- | |  | | NA | | NA |
| Mammary gland | +/+ | | -/+ | |  | | NA | | NA |
| Ovary (left) | +/- | | -/- | |  | | NA | | NA |

NA: not applicable. Grey-shaded areas: not done due to lack of material
